# Supplementary material for: A Delphi study to explore and gain consensus regarding the most important barriers and facilitators affecting physiotherapist and pharmacist non-medical prescribing
Source: PLoS One. 2021 Feb 2;16(2):e0246273. doi: 10.1371/journal.pone.0246273 (PMC7853445; doi:10.1371/journal.pone.0246273)
Supplement: S2 Appendix — (PDF) [file pone.0246273.s002.pdf]

Delphi Round 1 - NMP

Introduction

Thank you for agreeing to participate in this study, which is investigating barriers and facilitators to prescribing experienced by pharmacist and physiotherapist independent prescribers.

In this questionnaire, you will first be asked a few general questions about yourself. You will then be asked to list barriers and facilitators to prescribing that you have experienced.

- You will see a progress bar at the top of the page and this will indicate how far through the questionnaire you are.
- The questionnaire will take on average 30 minutes to complete but this may vary depending on how much you wish to write.
- You will be able to go back and forwards through the questionnaire if you need to.
- You will also be able to save the questionnaire and come back and finish it at a later time. To do this you will need to carefully follow the instructions that will be given at the time.

If you have any questions or comments please do not hesitate to contact me:

Emma Graham-Clarke, email address: EMG315@bham.ac.uk

I consent to participate in this study ✱ *Required*

○ Yes ○ No

Demographic data

About you

Which profession do you belong to? ✱ *Required*

- ○ Pharmacist
- ○ Physiotherapist

Which is the main area that you practice in? ✱ *Required*

- ○ Primary Care
- ○ Secondary care
- ○ Community
- ○ Other

If you selected Other, please specify:

____

Do you practice in any other areas? *Optional*

- ☐ Primary Care
- ☐ Secondary care
- ☐ Community
- ☐ Other

If you selected Other, please specify:

How many years have you been qualified in your profession? ✱ *Required*

- ≥5
- 6-10
- 11-15
- 16-20
- >21

About your prescribing

Which home nation did you qualify as a prescriber in? ✱ *Required*

- England
- Scotland
- Wales
- Northern Ireland

How long have you been qualified as an independent prescriber? ✱ *Required*

- ≤ 12 months
- > 12 months

Are you actively prescribing? ✱ *Required*

- ○ Yes
- ○ No

If yes, how many prescriptions on average do you write a week?

- ○ <5
- ○ 6-15
- ○ 16-25
- ○ 26-35
- ○ 36-45
- ○ >46

How would you describe your area of prescribing? *Optional*

- ○ I would describe myself as a specialist
- ○ I would describe myself as a generalist

If you describe yourself as a specialist, then please describe your specialty

Facilitators and barriers

You will now be asked to list any barriers or facilitators to non-medical prescribing that you have encountered.

- A **facilitator** is something that has helped, or made it easier, for you to practice as a prescriber.
- A **barrier** is something that has prevented, or made it difficult, for you to practice as a prescriber.

Please list at least three barriers and/or facilitators. You can list as many as you wish. Please use the free text box to add any information concerning the facilitator and/or barrier. For example, why you think it is important or how it has affected your practice.

Facilitator/Barrier * *Required*

More info

Would you describe this as a facilitator or barrier? * *Required*

- ○ Facilitator
- ○ Barrier

Free text box for comments

Facilitator/Barrier * *Required*

More info

Would you describe this as a facilitator or barrier? ✱ *Required*

◯ Facilitator ◯ Barrier

Free text box for comments

Facilitator/Barrier ✱ *Required*

More info

Would you describe this as a facilitator or barrier? ✱ *Required*

◯ Facilitator ◯ Barrier

Free text box for comments

Facilitator/Barrier *Optional*

More info

Would you describe this as a facilitator or barrier?

- ○ Facilitator
- ○ Barrier

Free text box for comments

Do you wish to add more facilitators and/or barriers? ✱ *Required*

- ○ Yes
- ○ No

Extra Facilitators and Barriers

Facilitator/Barrier *Optional*

More info

Would you describe this as a facilitator or barrier?

- Facilitator
- Barrier

Free text box for comments

Facilitator/Barrier *Optional*

More info

Would you describe this as a facilitator or barrier?

- Facilitator
- Barrier

Free text box for comments

Do you wish to add more facilitators and/or barriers? ✱ *Required*

- Yes
- No

Extra Facilitators and Barriers

Facilitator/Barrier *Optional*

More info

Would you describe this as a facilitator or barrier?

- Facilitator
- Barrier

Free text box for comments

Facilitator/Barrier *Optional*

More info

Would you describe this as a facilitator or barrier?

- Facilitator
- Barrier

Free text box for comments

Do you wish to add more facilitators and/or barriers? ✱ *Required*

- ○ Yes
- ○ No

Extra Facilitators and Barriers

Facilitator/Barrier *Optional*

More info

Would you describe this as a facilitator or barrier?

- Facilitator
- Barrier

Free text box for comments

Facilitator/Barrier *Optional*

More info

Would you describe this as a facilitator or barrier?

- Facilitator
- Barrier

Free text box for comments

Do you wish to add more facilitators and/or barriers? ✱ *Required*

- ◯ Yes
- ◯ No

Extra Facilitators and Barriers

Facilitator/Barrier *Optional*

More info

Would you describe this as a facilitator or barrier?

- Facilitator
- Barrier

Free text box for comments

Facilitator/Barrier *Optional*

More info

Would you describe this as a facilitator or barrier?

- Facilitator
- Barrier

Free text box for comments

Do you wish to add more facilitators and/or barriers? ✱ *Required*

- ◯ Yes
- ◯ No

Extra Facilitators and Barriers

Facilitator/Barrier *Optional*

More info

Would you describe this as a facilitator or barrier?

- Facilitator
- Barrier

Free text box for comments

Facilitator/Barrier *Optional*

More info

Would you describe this as a facilitator or barrier?

- Facilitator
- Barrier

Free text box for comments

Do you wish to add more facilitators and/or barriers? ✱ *Required*

- ◯ Yes
- ◯ No

Extra Facilitators and Barriers

Facilitator/Barrier *Optional*

More info

Would you describe this as a facilitator or barrier?

- Facilitator
- Barrier

Free text box for comments

Facilitator/Barrier *Optional*

More info

Would you describe this as a facilitator or barrier?

- Facilitator
- Barrier

Free text box for comments

Do you wish to add more facilitators and/or barriers? ✱ *Required*

- Yes
- No

Extra Facilitators and Barriers

Facilitator/Barrier *Optional*

More info

Would you describe this as a facilitator or barrier?

Facilitator Barrier

Free text box for comments

Facilitator/Barrier *Optional*

More info

Would you describe this as a facilitator or barrier?

Facilitator Barrier

Free text box for comments

Additional comments

Do you have any comments about completing this questionnaire, such as ease of completion?

The next steps:

All the responses will be reviewed to identify common themes and used to produce a list of the facilitators and barriers that have affected the participants.

You will then be sent a link to a new questionnaire. In the next questionnaire, you will be asked to review the list of barriers and facilitators and decide which of these you consider to be important.

The next questionnaire will be sent out in approximately six weeks from: 15 October 2018

Thank you

Thank you for completing this questionnaire.

If you have any questions or comments please do not hesitate to contact the lead researcher:

Emma Graham-Clarke, email address: EMG315@bham.ac.uk
