# Supplementary material for: A Delphi study to explore and gain consensus regarding the most important barriers and facilitators affecting physiotherapist and pharmacist non-medical prescribing
Source: PLoS One. 2021 Feb 2;16(2):e0246273. doi: 10.1371/journal.pone.0246273 (PMC7853445; doi:10.1371/journal.pone.0246273)
Supplement: S3 Appendix — (PDF) [file pone.0246273.s003.pdf]

Delphi Round 2 - NMP

Page 1: Introduction

Thank you for agreeing to participate in this study, which is to investigate barriers and facilitators to prescribing experienced by pharmacist and physiotherapist independent prescribers.

In this questionnaire you will be presented with various statements, developed from the responses to the last questionnaire that you participated in. Information in [ ] clarifies a statement, or makes it applicable to all areas of work and professions.

The statements have been divided into two sections; the first lists the various facilitators to non-medical prescribing that you have described, and the second lists the various barriers that you have also described. Each section comprises six tables of statements, grouped approximately by theme.

You will be asked to rate, using a simple scale, whether or not you agree that these are important factors. You will be able to add any comments you wish regarding each statement, such as elaborating on why you have chosen the rating you have given. You will also have the opportunity to add any further comments you may have.

- You will see a progress bar at the top of the page and this will indicate how far through the questionnaire you are.
- The questionnaire will take on average 30 minutes to complete but this may vary depending on how much you wish to write.
- You will be able to go back and forwards through the questionnaire if you need to.
- You will also be able to save the questionnaire and come back and finish it at a later time. To do this you will need to carefully follow the instructions that will be given at the time.

If you have any questions or comments please do not hesitate to contact me: Emma Graham-Clarke, email address: EMG315@bham.ac.uk

Page 2: Rating of facilitators

1. Please rate the following facilitator statements for their importance to you and your prescribing practice. Please use the free text box to add any comments elaborating on your choice of rating for that statement. A **facilitator** is defined as something that has helped, or made it easier, for you to practice as a prescriber.

	Rating ✱ *Required*					
	Strongly disagree (1)	Disagree (2)	Neutral (3)	Agree (4)	Strongly agree (5)	*Free text comments*
Management support enables funding and training time to qualify as a prescriber	☐	☐	☐	☐	☐	
Manager prompting [me] to do the course and plan how to introduce it in the department.	☐	☐	☐	☐	☐	
My employer has provided the support for me to be able to go on the NMP course and then supported me once qualified	☐	☐	☐	☐	☐	
My manager is keen to develop non-medical prescribers within the trust so is supportive of my role and helping me to negotiate a clinic slot again.	☐	☐	☐	☐	☐	
Support from my line manager	☐	☐	☐	☐	☐	
Support from the employer/department for the role of non-medical prescribers	☐	☐	☐	☐	☐	
The department created a role that allows me to utilise my skillset and supports me in the role	☐	☐	☐	☐	☐	

All patients rated the NMP experience as high and highly value their NMP prescribing as part of their care	☐	☐	☐	☐	☐	
Patient requirements. A need for patient's to have streamlined care by being able to prescribe at the point of contact	☐	☐	☐	☐	☐	
Being able to prescribe to patients is more effective and really useful working [in my area]	☐	☐	☐	☐	☐	
Wide variety of options that you can offer patients to improve their experience	☐	☐	☐	☐	☐	
Motivation to help the patients who will benefit with prescribing and cut care delay / duplication	☐	☐	☐	☐	☐	

1.a. Would you change the wording of any of the above statements? If you would, please describe say what you would change in the following free text box.

2. Please rate the following facilitator statements for their importance to you and your prescribing practice. Please use the free text box to add any comments elaborating on your choice of rating for that statement. A **facilitator** is defined as something that has helped, or made it easier, for you to practice as a prescriber.

	Rating ✱ *Required*					
	Strongly disagree (1)	Disagree (2)	Neutral (3)	Agree (4)	Strongly agree (5)	*Free text comments*

Medical colleagues informed by my frequent prescribing habits and have begun prescribing common drugs I often start a patient on	☐	☐	☐	☐	☐	
Clinical supervision with a [doctor] has massively helped me increase my confidence prescribing	☐	☐	☐	☐	☐	
Direct contact with medical team caring for patient	☐	☐	☐	☐	☐	
Doctors have been working [with] this [NMP] model	☐	☐	☐	☐	☐	
Forward thinking DMP [designated medical practitioner] who is keen to integrate different MDG [multidisciplinary group] professionals into the team	☐	☐	☐	☐	☐	
Good relationship with consultants	☐	☐	☐	☐	☐	
Joint working / shadowing opportunities with the specialist prescribers or GPs	☐	☐	☐	☐	☐	

Supportive medical supervision / mentorship	☐	☐	☐	☐	☐	
Supportive medical colleagues	☐	☐	☐	☐	☐	
Supportive nursing colleagues	☐	☐	☐	☐	☐	

2.a. Would you change the wording of any of the above statements? If you would, please describe say what you would change in the following free text box.

3. Please rate the following facilitator statements for their importance to you and your prescribing practice. Please use the free text box to add any comments elaborating on your choice of rating for that statement. A **facilitator** is defined as something that has helped, or made it easier, for you to practice as a prescriber.

	Rating ✱ *Required*					
	Strongly disagree (1)	Disagree (2)	Neutral (3)	Agree (4)	Strongly agree (5)	*Free text comments*
The nature of the role facilitates prescribing practice as part of the overall review of patients	☐	☐	☐	☐	☐	
Mentor already NMP - creates a positive environment for NMP	☐	☐	☐	☐	☐	
Being a role model for others [of my profession] as the only in-patient NMP	☐	☐	☐	☐	☐	

My knowledge of medication	☐	☐	☐	☐	☐	
As an NMP I have much better knowledge of OTC [over the counter] medication and can advise patients accordingly	☐	☐	☐	☐	☐	
My experience working as alongside a consultant/[GP] for many years	☐	☐	☐	☐	☐	
Having a speciality allows development of skills and knowledge	☐	☐	☐	☐	☐	
Evidence base from investigations	☐	☐	☐	☐	☐	
Prescribing regularly in primary care, a most advantageous skill	☐	☐	☐	☐	☐	
30 years experience [giving confidence]	☐	☐	☐	☐	☐	
Personal confidence in specialism	☐	☐	☐	☐	☐	

3.a. Would you change the wording of any of the above statements? If you would, please describe say what you would change in the following free text box.

4. Please rate the following facilitator statements for their importance to you and your prescribing practice. Please use the free text box to add any comments elaborating on your choice of rating for that statement. A **facilitator** is defined as something that has helped, or made it easier, for you to practice as a prescriber.

	Rating ✱ *Required*					
	Strongly disagree (1)	Disagree (2)	Neutral (3)	Agree (4)	Strongly agree (5)	*Free text comments*
Clinical Lead pushing the project forwards	☐	☐	☐	☐	☐	
Good NMP support group with regular meetings	☐	☐	☐	☐	☐	
Working as part of an MDT [multidisciplinary team] / interdisciplinary group	☐	☐	☐	☐	☐	
Nursing and medical staff very open to pharmacist NMP role	☐	☐	☐	☐	☐	
Support from other NMPs	☐	☐	☐	☐	☐	
When you see others doing, I think it gives you the confidence to do it yourself	☐	☐	☐	☐	☐	
Well supported by team and they allow me to prescribe for their patients	☐	☐	☐	☐	☐	

4.a. Would you change the wording of any of the above statements? If you would, please describe say what you would change in the following free text box.

5. Please rate the following facilitator statements for their importance to you and your prescribing practice. Please use the free text box to add any comments elaborating on your choice of rating for that statement. A **facilitator** is defined as something that has helped, or made it easier, for you to practice as a prescriber.

	Rating ✱ *Required*					
	Strongly disagree (1)	Disagree (2)	Neutral (3)	Agree (4)	Strongly agree (5)	*Free text comments*
Process for registering, getting prescription pads etc... in place	☐	☐	☐	☐	☐	
Room space available for clinics	☐	☐	☐	☐	☐	
The law enables me to practice as an NMP	☐	☐	☐	☐	☐	
As an IP [independent prescriber] it has made doing steroid injections much easier as less paperwork is necessary	☐	☐	☐	☐	☐	

As an IP [independent prescriber] it has made the process of steroid injections easier as I am able to mix medication	☐	☐	☐	☐	☐	
Electronic prescribing system allows an audit trail of my prescribing and pharmacists can easily access the prescriptions and verify them	☐	☐	☐	☐	☐	
Having an electronic patient record mean that I can use all patient data available to base my prescribing upon	☐	☐	☐	☐	☐	
I have a [prescribing] budget where I work	☐	☐	☐	☐	☐	
Lack of medical cover proves the need have an extra prescriber on the ward	☐	☐	☐	☐	☐	
Nurses are not yet prescribers	☐	☐	☐	☐	☐	

5.a. Would you change the wording of any of the above statements? If you would, please describe say what you would change in the following free text box.

6. Please rate the following facilitator statements for their importance to you and your prescribing practice. Please use the free text box to add any comments elaborating on your choice of rating for that statement. A **facilitator** is defined as something that has helped, or made it easier, for you to practice as a prescriber.

	Rating ✱ *Required*					
	Strongly disagree (1)	Disagree (2)	Neutral (3)	Agree (4)	Strongly agree (5)	*Free text comments*
Supportive pharmacy leadership allowing prescribing without insisting on a second check by pharmacist	☐	☐	☐	☐	☐	
[Benefit of] NICE Guidelines	☐	☐	☐	☐	☐	
Great antibiotic guidelines in this trust/area	☐	☐	☐	☐	☐	
Easy access to medication info	☐	☐	☐	☐	☐	
Supportive working environment [with NMP] policies in place	☐	☐	☐	☐	☐	
We are well supported with NMP training opportunities, including 2 full in-house training days a year	☐	☐	☐	☐	☐	

Effective personal development reviews	☐	☐	☐	☐	☐	
Ongoing mentorship [supports] CPD [continuing professional development] and keeping up to date with current medication regimes	☐	☐	☐	☐	☐	
Attendance of MDT [multidisciplinary team] meeting [as] patients are discussed allowing the prescription to be discussed with the team	☐	☐	☐	☐	☐	

6.a. Would you change the wording of any of the above statements? If you would, please describe say what you would change in the following free text box.

7. Do you feel that there are any facilitators that are missing? If there are, please add them to the following free text box.

Page 3: Rating of barriers

8. Please rate the following barrier statements for their importance to you and your prescribing practice. Please use the free text box to add any comments elaborating on your choice of rating for that statement. A **barrier** is defined as something that has prevented, or made it difficult, for you to practice as a prescriber.

	Rating ✱ *Required*					
	Strongly disagree (1)	Disagree (2)	Neutral (3)	Agree (4)	Strongly agree (5)	*Free text comments*
I am starting a new service, without much peer/managerial support to set it up	☐	☐	☐	☐	☐	
Managers not supporting prescribing role	☐	☐	☐	☐	☐	
The department is not very supportive within the context of expanding my role and utilising the practical aspects of my prescribing such as patient examination	☐	☐	☐	☐	☐	
Trust application process lengthy and still waiting to be allowed to prescribe. Director of nursing does not know me and has been reluctant to sign paperwork	☐	☐	☐	☐	☐	
[Lack of] acceptance as NMP by nurses	☐	☐	☐	☐	☐	

Pressure from ward to prescribe beyond my scope	☐	☐	☐	☐	☐	
A lack of clinicians wanting to share their skills	☐	☐	☐	☐	☐	
Colleagues may feel prescribing should only occur after all the usual duties have been completed	☐	☐	☐	☐	☐	
I have no other IP [independent prescriber] to chat things through with quickly & easily	☐	☐	☐	☐	☐	
Lack of staffing, so often conducting medicines reconciliation, which I am reluctant to prescribe from, and may not have time to go on the ward round	☐	☐	☐	☐	☐	
Sometimes junior clinicians feel an NMP is prescribing because their own prescribing is inadequate	☐	☐	☐	☐	☐	

8.a. Would you change the wording of any of the above statements? If you would, please describe say what you would change in the following free text box.

9. Please rate the following barrier statements for their importance to you and your prescribing practice. Please use the free text box to add any comments elaborating on your choice of rating for that statement. A **barrier** is defined as something that has prevented, or made it difficult, for you to practice as a prescriber.

	Rating ✱ *Required*					
	Strongly disagree (1)	Disagree (2)	Neutral (3)	Agree (4)	Strongly agree (5)	*Free text comments*
[Medic] instead of increasing the dose may change the drug instead of discussing with me	☐	☐	☐	☐	☐	
Lack of acceptance by [medics]	☐	☐	☐	☐	☐	
Lack of immediate medical advice/support	☐	☐	☐	☐	☐	
Lack of suitable mentor/mentorship	☐	☐	☐	☐	☐	
Non-attendance on clinical ward rounds as documentation of patient progress or clinical plan in notes not always clear	☐	☐	☐	☐	☐	
Community/lone working [as] we only ever see patients on our own, so I'm unable to gain advice from other sources immediately	☐	☐	☐	☐	☐	
Poor integration between the community team and the hospital team	☐	☐	☐	☐	☐	

Secondary care outpatient specialities should suggest course of action to the GP regarding medicines. (Clinical Pathways and Hospital Policies)	☐	☐	☐	☐	☐	
New ways of working from joining new team	☐	☐	☐	☐	☐	
I am unable to access any shared medical records making prescribing very difficult	☐	☐	☐	☐	☐	

9.a. Would you change the wording of any of the above statements? If you would, please describe say what you would change in the following free text box.

10. Please rate the following barrier statements for their importance to you and your prescribing practice. Please use the free text box to add any comments elaborating on your choice of rating for that statement. A **barrier** is defined as something that has prevented, or made it difficult, for you to practice as a prescriber.

	Rating ✱ *Required*					
	Strongly disagree (1)	Disagree (2)	Neutral (3)	Agree (4)	Strongly agree (5)	*Free text comments*
Lacking confidence as it is a new skill and not enough exposure	☐	☐	☐	☐	☐	

My confidence. I do sometimes doubt my abilities and worry a great deal about the legal/professional implications of making an incorrect decision	☐	☐	☐	☐	☐	
Change in job role, [to one that] did not lend itself to prescribing	☐	☐	☐	☐	☐	
Changed roles [and] I don't feel confident to prescribe in the area that I work in now	☐	☐	☐	☐	☐	
Due to change employment shortly where new post doesn't currently have prescribing for [my profession] in place	☐	☐	☐	☐	☐	
NMP role not well established for [my profession]	☐	☐	☐	☐	☐	
No identified prescribing role in current work area	☐	☐	☐	☐	☐	
There is no time to actively prescribe [in my current role]	☐	☐	☐	☐	☐	
[Lack of] a defined reason to prescribe	☐	☐	☐	☐	☐	
Lack of clear requirements to what is competent	☐	☐	☐	☐	☐	

Formulary differences between Trust and APC/CCG [area prescribing committee/clinical commissioning group] make it difficult to know what I can prescribe	☐	☐	☐	☐	☐	

10.a. Would you change the wording of any of the above statements? If you would, please describe say what you would change in the following free text box.

11. Please rate the following barrier statements for their importance to you and your prescribing practice. Please use the free text box to add any comments elaborating on your choice of rating for that statement. A **barrier** is defined as something that has prevented, or made it difficult, for you to practice as a prescriber.

	Rating ✱ *Required*					*Free text comments*
	Strongly disagree (1)	Disagree (2)	Neutral (3)	Agree (4)	Strongly agree (5)	
Keeping up with new research difficult	☐	☐	☐	☐	☐	
You've had to stop a particular drug, even though licensed for reason being prescribed, as CCG [clinical commissioning group] is following guideline	☐	☐	☐	☐	☐	
Starting a new speciality with new medicines to learn about	☐	☐	☐	☐	☐	
Lack of diagnostic skills makes primary prescribing more difficult	☐	☐	☐	☐	☐	

Clinical examination skills. Only basics taught on the course - BP and pulse. This makes me apprehensive to prescribe	☐	☐	☐	☐	☐	
Lack of access to ongoing development out of Trust	☐	☐	☐	☐	☐	
Lack of training structure within the department[/workplace]	☐	☐	☐	☐	☐	
Lack of communication from university following course completion	☐	☐	☐	☐	☐	
Lack of pharmacology exposure during undergraduate training	☐	☐	☐	☐	☐	
NMP course very primary care and nursing focussed	☐	☐	☐	☐	☐	
Lack of clinic rooms	☐	☐	☐	☐	☐	
Rely on external company to register NMPs, print pads etc... sometimes delays someone being able to prescribe	☐	☐	☐	☐	☐	

11.a. Would you change the wording of any of the above statements? If you would, please describe say what you would change in the following free text box.

12. Please rate the following barrier statements for their importance to you and your prescribing practice. Please use the free text box to add any comments elaborating on your choice of rating for that statement. A **barrier** is defined as something that has prevented, or made it difficult, for you to practice as a prescriber.

	Rating ✱ *Required*					
	Strongly disagree (1)	Disagree (2)	Neutral (3)	Agree (4)	Strongly agree (5)	*Free text comments*
The availability of a pharmacist to clinically screen the prescriptions	☐	☐	☐	☐	☐	
[My] prescribing not reviewed by pharmacists in the same way as medic or other NMPs prescribing	☐	☐	☐	☐	☐	
Variable access to patient records. I would not be happy to prescribe when I did not have access to patient record with up to date medication/allergies etc.	☐	☐	☐	☐	☐	
Unable to access patient's full SCR [summary care records] / GP records	☐	☐	☐	☐	☐	
[Unable to prescribe certain drugs and have to use] supplementary prescribing, [which] requires a slight change to the pathway of the team and doctors need to be educated	☐	☐	☐	☐	☐	
Cost of professional indemnity	☐	☐	☐	☐	☐	
Lack of medical cover at times means I cannot prescribe opioids	☐	☐	☐	☐	☐	

Limitations of [legal] prescribing guidelines [with a disparity between practitioner roles]	☐	☐	☐	☐	☐	
Professional indemnity is a challenge to acquire - need updated JD [job description] and employer slow to produce	☐	☐	☐	☐	☐	
Skills learnt during NMP course cannot be put into practice until [professional] registration which took 2 months	☐	☐	☐	☐	☐	
Unable to prescribe [certain drugs] and have to ask a [doctor] to do this	☐	☐	☐	☐	☐	

12.a. Would you change the wording of any of the above statements? If you would, please describe say what you would change in the following free text box.

13. Please rate the following barrier statements for their importance to you and your prescribing practice. Please use the free text box to add any comments elaborating on your choice of rating for that statement. A **barrier** is defined as something that has prevented, or made it difficult, for you to practice as a prescriber.

	Rating ✱ *Required*					
	Strongly disagree (1)	Disagree (2)	Neutral (3)	Agree (4)	Strongly agree (5)	*Free text comments*
NMP in clinic is not recognised as not commissioned	☐	☐	☐	☐	☐	

[Lack of] Funding for time spent prescribing	☐	☐	☐	☐	☐	
Gaining permission for pilot and initial funding for clinics	☐	☐	☐	☐	☐	
Lack of organisational funding	☐	☐	☐	☐	☐	
No money to pay for staff going on any courses. However there seems to be a disparity as nurses in acute care appear to be able to access MSc modules	☐	☐	☐	☐	☐	
Nurses are cheaper	☐	☐	☐	☐	☐	
Nurse led clinic introducing nurse prescribers so no need for other prescribers	☐	☐	☐	☐	☐	
There is no current budget for prescribing	☐	☐	☐	☐	☐	

[Lack of time] time available for prescribing activities. Facilitating attendance on ward round to allow full patient history and inpatient episode history	☐	☐	☐	☐	☐	
[Lack of] time to specialise	☐	☐	☐	☐	☐	
Lack of allotted time resulting from new management role	☐	☐	☐	☐	☐	
Lack of time to develop further prescribing skills	☐	☐	☐	☐	☐	
Lack of time to prescribe as core/clinical duties take priority	☐	☐	☐	☐	☐	

13.a. Would you change the wording of any of the above statements? If you would, please describe say what you would change in the following free text box.

14. Do you feel that there are any barriers that are missing? If there are, please add them to the following free text box.

Page 4: Next Steps

15. Do you have any comments about completing this questionnaire, such as ease of completion?

The next steps:

All the responses from the questionnaire will be analysed, and some simple statistics calculated.

You will then be sent a link to a new questionnaire, which will enable you to compare your rating with the average panel result. If you wish to amend your response you will be able to do this. You will also be asked to rank the facilitators and barriers in order of importance.

The next questionnaire will be sent out in approximately six weeks from: 07/18

Page 5: Thank you

Thank you for completing this questionnaire.

If you have any questions or comments please do not hesitate to contact the lead researcher: Emma Graham-Clarke, email address: EMG315@bham.ac.uk
