# Supplementary material for: A Delphi study to explore and gain consensus regarding the most important barriers and facilitators affecting physiotherapist and pharmacist non-medical prescribing
Source: PLoS One. 2021 Feb 2;16(2):e0246273. doi: 10.1371/journal.pone.0246273 (PMC7853445; doi:10.1371/journal.pone.0246273)
Supplement: S4 Appendix — (PDF) [file pone.0246273.s004.pdf]

Delphi Round 3 - NMP

Page 1: Introduction

Thank you for continuing to participate in this study, which is to investigate barriers and facilitators to prescribing experienced by pharmacist and physiotherapist independent prescribers.

In this questionnaire you will have the opportunity to review ratings given to statements in the previous round, and to rank statements in order of their importance to you. Information in [ ] clarifies a statement, or makes it applicable to all areas of work and professions.

In the **first section** of the questionnaire you will be re-presented with statements from the last questionnaire that did not reach agreement on their importance.

By each statement you will see the median response that you and your fellow participants gave. For example: "Manager prompting [me] to do the course and plan how to introduce it in the department. (Median 4)"

Please reconsider your initial rating compared to the feedback you receive. If you wish to change the rating you gave to the statement, then you can do this using the rating scale. If you do not wish to amend your previous response, then leave the rating blank.

If the statement is not applicable to your practice, or you are unable to give an opinion on it, then please rate the statement as ‘neutral’.

You can add any further comments you wish regarding each statement, such as elaborating on why you have chosen the rating you have given, but you do not need to repeat any comments that you gave in the previous round.

Please remember that there are no right or wrong answers, I am seeking your opinions.

In the **second section** of the questionnaire you will have the opportunity to rank, in order of importance to you, those statements where consensus has already been achieved.

- You will see a progress bar at the top of the page, and this will indicate how far through the questionnaire you are.
- The questionnaire will take on average 30 minutes to complete but this may vary depending on how much you wish to write.
- You will be able to go back and forwards through the questionnaire if you need to.
- You will also be able to save the questionnaire and come back and finish it at a later time. To do this you will need to carefully follow the instructions that will be given at the time.

If you have any questions or comments please do not hesitate to contact me:

Emma Graham-Clarke, email address: EMG315@bham.ac.uk

Page 2: Review of facilitators

Please review the median rating assigned to each facilitator statement with regard to the importance to you and your prescribing practice and reconsider your initial rating. If you wish to change the rating you originally gave, then use the rating scale to do so. If you are happy with your original rating, then you need do nothing. If the statement is not applicable to you, or you have no opinion about it, then please rate it as neutral. Please use the free text box to add any comments elaborating on your rating for that statement.

More info

	Rating					
	Strongly disagree (1)	Disagree (2)	Neutral (3)	Agree (4)	Strongly agree (5)	*Free text comments*
All patients rated the NMP experience as high and highly value their NMP prescribing as part of their care (Median 4)	☐	☐	☐	☐	☐	
Clinical Lead pushing the project forwards (Median 4)	☐	☐	☐	☐	☐	
Good NMP support group with regular meetings (Median 4)	☐	☐	☐	☐	☐	
Joint working / shadowing opportunities with the specialist prescribers or GPs (Median 4)	☐	☐	☐	☐	☐	
Manager prompting [me] to do the course and plan how to introduce it in the department (Median 4)	☐	☐	☐	☐	☐	
Medical colleagues informed by my frequent prescribing habits and have begun prescribing common drugs I often start a patient on (Median 3)	☐	☐	☐	☐	☐	
My experience working as alongside a consultant/[GP] for many years (Median 4)	☐	☐	☐	☐	☐	
My manager is keen to develop non-medical prescribers within the trust so is supportive of my role and helping me to negotiate a clinic slot again (Median 4)	☐	☐	☐	☐	☐	

Nursing and medical staff very open to pharmacist NMP role (Median 4)	☐	☐	☐	☐	☐	
Supportive pharmacy leadership allowing prescribing without insisting on a second check by pharmacist (Median 4)	☐	☐	☐	☐	☐	

Please review the median rating assigned to each facilitator statement with regard to the importance to you and your prescribing practice and reconsider your initial rating. If you wish to change the rating you originally gave, then use the rating scale to do so. If you are happy with your original rating, then you need do nothing. If the statement is not applicable to you, or you have no opinion about it, then please rate it as neutral. Please use the free text box to add any comments elaborating on your rating for that statement.

More info

	Rating					*Free text comments*
	Strongly disagree (1)	Disagree (2)	Neutral (3)	Agree (4)	Strongly agree (5)	
As an NMP I have much better knowledge of OTC [over the counter] medication and can advise patients accordingly (Median 4)	☐	☐	☐	☐	☐	
Attendance of MDT [multidisciplinary team] meeting [as] patients are discussed allowing the prescription to be discussed with the team (Median 4)	☐	☐	☐	☐	☐	
[Benefit of] NICE Guidelines (Median 4)	☐	☐	☐	☐	☐	
Effective personal development reviews (Median 4)	☐	☐	☐	☐	☐	
Evidence base from investigations (Median 4)	☐	☐	☐	☐	☐	
Having an electronic patient record mean that I can use all patient data available to base my prescribing upon (Median 4)	☐	☐	☐	☐	☐	

Ongoing mentorship [supports] CPD [continuing professional development] and keeping up to date with current medication regimes (Median 4)	☐	☐	☐	☐	☐	
Prescribing regularly in primary care, a most advantageous skill (Median 3)	☐	☐	☐	☐	☐	
Process for registering, getting prescription pads etc... in place (Median 3)	☐	☐	☐	☐	☐	
We are well supported with NMP training opportunities, including 2 full in-house training days a year (Median 3)	☐	☐	☐	☐	☐	
When you see others doing, I think it gives you the confidence to do it yourself (Median 4)	☐	☐	☐	☐	☐	

Page 3: Review of barriers

Please review the median rating assigned to each barrier statement with regard to the importance to you and your prescribing practice and reconsider your initial rating. If you wish to change the rating you originally gave, then use the rating scale to do so. If you are happy with your original rating, then you need do nothing. If the statement is not applicable to you, or you have no opinion about it, then please rate it as neutral. Please use the free text box to add any comments elaborating on your rating for that statement.

More info

	Rating					
	Strongly disagree (1)	Disagree (2)	Neutral (3)	Agree (4)	Strongly agree (5)	*Free text comments*
A lack of clinicians wanting to share their skills (Median 2)	☐	☐	☐	☐	☐	
Colleagues may feel prescribing should only occur after all the usual duties have been completed (Median 2)	☐	☐	☐	☐	☐	
I am starting a new service, without much peer/managerial support to set it up (Median 3)	☐	☐	☐	☐	☐	
I have no other IP [independent prescriber] to chat things through with quickly & easily (Median 1)	☐	☐	☐	☐	☐	
Lack of immediate medical advice/support (Median 2)	☐	☐	☐	☐	☐	
New ways of working from joining new team (Median 3)	☐	☐	☐	☐	☐	
Poor integration between the community team and the hospital team (Median 3)	☐	☐	☐	☐	☐	
Sometimes junior clinicians feel an NMP is prescribing because their own prescribing is inadequate (Median 3)	☐	☐	☐	☐	☐	

The department is not very supportive within the context of expanding my role and utilising the practical aspects of my prescribing such as patient examination (Median 2)	☐	☐	☐	☐	☐	

Please review the median rating assigned to each barrier statement with regard to the importance to you and your prescribing practice and reconsider your initial rating. If you wish to change the rating you originally gave, then use the rating scale to do so. If you are happy with your original rating, then you need do nothing. If the statement is not applicable to you, or you have no opinion about it, then please rate it as neutral. Please use the free text box to add any comments elaborating on your rating for that statement.

More info

	Rating					
	Strongly disagree (1)	Disagree (2)	Neutral (3)	Agree (4)	Strongly agree (5)	*Free text comments*
[Lack of] a defined reason to prescribe (Median 2)	☐	☐	☐	☐	☐	
Lack of access to ongoing development out of Trust (Median 2)	☐	☐	☐	☐	☐	
Lack of communication from university following course completion (Median 2)	☐	☐	☐	☐	☐	
Lacking confidence as it is a new skill and not enough exposure (Median 4)	☐	☐	☐	☐	☐	
Lack of diagnostic skills makes primary prescribing more difficult (Median 3)	☐	☐	☐	☐	☐	
Lack of pharmacology exposure during undergraduate training (Median 2)	☐	☐	☐	☐	☐	
Lack of training structure within the department[/workplace] (Median 3)	☐	☐	☐	☐	☐	

My confidence. I do sometimes doubt my abilities and worry a great deal about the legal/professional implications of making an incorrect decision (Median 4)	☐	☐	☐	☐	☐	
NMP role not well established for [my profession] (Median 2)	☐	☐	☐	☐	☐	
Starting a new speciality with new medicines to learn about (Median 3)	☐	☐	☐	☐	☐	

Page 4: Review of barriers (part 2)

Please review the median rating assigned to each barrier statement with regard to the importance to you and your prescribing practice and reconsider your initial rating. If you wish to change the rating you originally gave, then use the rating scale to do so. If you are happy with your original rating, then you need do nothing. If the statement is not applicable to you, or you have no opinion about it, then please rate it as neutral. Please use the free text box to add any comments elaborating on your rating for that statement.

More info

	Rating					
	Strongly disagree (1)	Disagree (2)	Neutral (3)	Agree (4)	Strongly agree (5)	*Free text comments*
Cost of professional indemnity (Median 2)	☐	☐	☐	☐	☐	
Lack of clinic rooms (Median 2)	☐	☐	☐	☐	☐	
Lack of medical cover at times means I cannot prescribe opioids (Median 3)	☐	☐	☐	☐	☐	
Limitations of [legal] prescribing guidelines [with a disparity between practitioner roles] (Median 3)	☐	☐	☐	☐	☐	
[My] prescribing not reviewed by pharmacists in the same way as medic or other NMPs prescribing (Median 2)	☐	☐	☐	☐	☐	
The availability of a pharmacist to clinically screen the prescriptions (Median 2)	☐	☐	☐	☐	☐	
[Unable to prescribe certain drugs and have to use] supplementary prescribing, [which] requires a slight change to the pathway of the team and doctors need to be educated (Median 2)	☐	☐	☐	☐	☐	
Variable access to patient records. I would not be happy to prescribe when I did not have access to patient record with up to date medication/allergies etc. (Median 3)	☐	☐	☐	☐	☐	

Please review the median rating assigned to each barrier statement with regard to the importance to you and your prescribing practice and reconsider your initial rating. If you wish to change the rating you originally gave, then use the rating scale to do so. If you are happy with your original rating, then you need do nothing. If the statement is not applicable to you, or you have no opinion about it, then please rate it as neutral. Please use the free text box to add any comments elaborating on your rating for that statement.

More info

	Rating					
	Strongly disagree (1)	Disagree (2)	Neutral (3)	Agree (4)	Strongly agree (5)	*Free text comments*
Lack of allotted time resulting from new management role (Median 3)	☐	☐	☐	☐	☐	
Lack of organisational funding (Median 2)	☐	☐	☐	☐	☐	
[Lack of] time available for prescribing activities. Facilitating attendance on ward round to allow full patient history and inpatient episode history (Median 3)	☐	☐	☐	☐	☐	
Lack of time to prescribe as core/clinical duties take priority (Median 4)	☐	☐	☐	☐	☐	
[Lack of] time to specialise (Median 2)	☐	☐	☐	☐	☐	
Nurses are cheaper (Median 3)	☐	☐	☐	☐	☐	
Nurse led clinic introducing nurse prescribers so no need for other prescribers (Median 3)	☐	☐	☐	☐	☐	
Professional indemnity is a challenge to acquire - need updated JD [job description] and employer slow to produce (Median 3)	☐	☐	☐	☐	☐	
Skills learnt during NMP course cannot be put into practice until [professional] registration which took 2 months (Median 4)	☐	☐	☐	☐	☐	

Unable to prescribe [certain drugs] and have to ask a [doctor] to do this (Median 3)	☐	☐	☐	☐	☐	

Page 5: Ranking the statements

Below are a number of statements that you were asked to rate in the previous questionnaire, and which achieved consensus at that stage. Please select the 10 most important statements that affect your practice and rank them from 1 to 10 (where 1 is most important and 10 least important). ✱ *Required*

Please don't select more than 1 answer(s) per row.

Please select between 5 and 10 answers.

Please don't select more than 1 answer(s) in any single column.

	1	2	3	4	5	6	7	8	9	10
Being able to prescribe to patients is more effective and really useful working [in my area]	☐	☐	☐	☐	☐	☐	☐	☐	☐	☐
Clinical supervision with a [doctor] has massively helped me increase my confidence prescribing	☐	☐	☐	☐	☐	☐	☐	☐	☐	☐
Doctors have been working [with] this [NMP] model	☐	☐	☐	☐	☐	☐	☐	☐	☐	☐
Direct contact with medical team caring for patient	☐	☐	☐	☐	☐	☐	☐	☐	☐	☐
Easy access to medication info	☐	☐	☐	☐	☐	☐	☐	☐	☐	☐
Forward thinking DMP [designated medical practitioner] who is keen to integrate different MDG [multidisciplinary group] professionals into the team	☐	☐	☐	☐	☐	☐	☐	☐	☐	☐
Great antibiotic guidelines in this trust/area	☐	☐	☐	☐	☐	☐	☐	☐	☐	☐
Good relationship with consultants	☐	☐	☐	☐	☐	☐	☐	☐	☐	☐
Having a speciality allows development of skills and knowledge	☐	☐	☐	☐	☐	☐	☐	☐	☐	☐
Lack of time to develop further prescribing skills	☐	☐	☐	☐	☐	☐	☐	☐	☐	☐

Management support enables funding and training time to qualify as a prescriber	☐	☐	☐	☐	☐	☐	☐	☐	☐	☐
My knowledge of medication	☐	☐	☐	☐	☐	☐	☐	☐	☐	☐
My employer has provided the support for me to be able to go on the NMP course and then supported me once qualified	☐	☐	☐	☐	☐	☐	☐	☐	☐	☐
Mentor already NMP - creates a positive environment for NMP	☐	☐	☐	☐	☐	☐	☐	☐	☐	☐
Motivation to help the patients who will benefit with prescribing and cut care delay / duplication	☐	☐	☐	☐	☐	☐	☐	☐	☐	☐
Personal confidence in specialism	☐	☐	☐	☐	☐	☐	☐	☐	☐	☐
Patient requirements. A need for patients to have streamlined care by being able to prescribe at the point of contact	☐	☐	☐	☐	☐	☐	☐	☐	☐	☐
Supportive medical colleagues	☐	☐	☐	☐	☐	☐	☐	☐	☐	☐
Supportive medical supervision / mentorship	☐	☐	☐	☐	☐	☐	☐	☐	☐	☐
Supportive nursing colleagues	☐	☐	☐	☐	☐	☐	☐	☐	☐	☐
Supportive working environment [with NMP] policies in place	☐	☐	☐	☐	☐	☐	☐	☐	☐	☐
Support from my line manager	☐	☐	☐	☐	☐	☐	☐	☐	☐	☐
Support from the employer/department for the role of non-medical prescribers	☐	☐	☐	☐	☐	☐	☐	☐	☐	☐

Support from other NMPs	☐	☐	☐	☐	☐	☐	☐	☐	☐	☐
The law enables me to practice as an NMP	☐	☐	☐	☐	☐	☐	☐	☐	☐	☐
The nature of the role facilitates prescribing practice as part of the overall review of patients	☐	☐	☐	☐	☐	☐	☐	☐	☐	☐
Wide variety of options that you can offer patients to improve their experience	☐	☐	☐	☐	☐	☐	☐	☐	☐	☐
Well supported by team and they allow me to prescribe for their patients	☐	☐	☐	☐	☐	☐	☐	☐	☐	☐
Working as part of an MDT [multidisciplinary team] / interdisciplinary group	☐	☐	☐	☐	☐	☐	☐	☐	☐	☐

Page 6: Next Steps

Do you have any comments about completing this questionnaire, such as ease of completion?

Thank you for your participation in this doctoral research study. If you would like to be informed of the research results, and receive a copy of the final results, then please give your email address below.

Please enter a valid email address.

Page 7: Thank you

Thank you for completing this questionnaire.
If you have any questions or comments please do not hesitate to contact the lead researcher: Emma Graham-Clarke, email address: EMG315@bham.ac.uk

---
