# Supplementary material for: A Delphi study to explore and gain consensus regarding the most important barriers and facilitators affecting physiotherapist and pharmacist non-medical prescribing
Source: PLoS One. 2021 Feb 2;16(2):e0246273. doi: 10.1371/journal.pone.0246273 (PMC7853445; doi:10.1371/journal.pone.0246273)
Supplement: S5 Appendix — (DOCX) [file pone.0246273.s005.docx]

**Weighted rank sum – worked example for statement**: “Being able to prescribe to patients is more effective and really useful working [in my area]”

Step 1 – reverse the rank. For example, rank 1 becomes 10

Step 2 – count the number at each rank – this is the weighting. In this example 9 participants have ranked the statement at 10, 3 at 9, etc.

Step 3 – multiply the reversed rank by the weighting

| **Original rank** | **Reverse rank** | **Weighting** | **Weighted rank** |
| --- | --- | --- | --- |
| 3 | 8 | 1 | 8 |
| 1 | 10 | 9 | 90 |
| 10 | 1 | 1 | 1 |
| 2 | 9 | 3 | 27 |
| 2 | 9 | 3 | 27 |
| 1 | 10 | 9 | 90 |
| 6 | 5 | 1 | 5 |
| 1 | 10 | 9 | 90 |
| 1 | 10 | 9 | 90 |
| 8 | 3 | 2 | 6 |
| 1 | 10 | 9 | 90 |
| 2 | 9 | 3 | 27 |
| 1 | 10 | 9 | 90 |
| 1 | 10 | 9 | 90 |
| 1 | 10 | 9 | 90 |
| 8 | 3 | 2 | 6 |
| 1 | 10 | 9 | 90 |
