# Supplementary material for: A Delphi study to explore and gain consensus regarding the most important barriers and facilitators affecting physiotherapist and pharmacist non-medical prescribing
Source: PLoS One. 2021 Feb 2;16(2):e0246273. doi: 10.1371/journal.pone.0246273 (PMC7853445; doi:10.1371/journal.pone.0246273)
Supplement: S1 Table — (DOCX) [file pone.0246273.s006.docx]

### S1 Table. Sample matrix for selecting Delphi participants

| Criteria | Pharmacist | Physiotherapist | Years of professional practice | |
| --- | --- | --- | --- | --- |
| Profession | 30 | 30 |  |  |
| Length of time qualified as a prescriber: | | | ≥5 | 0-10 |
| ≥12 months | 5-20 | 5-20 | 6-10 | 0-10 |
| <12 months | 5-20 | 5-20 | 11-15 | 0-10 |
| Main practice area: | | | 16-20 | 0-10 |
| Primary Care | 5-20 | 5-20 | >21 | 0-10 |
| Secondary care | 5-20 | 5-20 |  |  |
| Community | 0-5 | 0-5 |  |  |
| Other (please describe) | 0-5 | 0-5 |  |  |
| Other requested information (but no control required for sampling):  Home nation – England, Scotland, Wales, Northern Ireland | | | | |
