# Supplementary material for: A Delphi study to explore and gain consensus regarding the most important barriers and facilitators affecting physiotherapist and pharmacist non-medical prescribing
Source: PLoS One. 2021 Feb 2;16(2):e0246273. doi: 10.1371/journal.pone.0246273 (PMC7853445; doi:10.1371/journal.pone.0246273)
Supplement: S2 Table — (DOCX) [file pone.0246273.s007.docx]

### S2 Table. Consensus results for Facilitator statements, Round Two – grouped by all participants and for each profession

Key to S2 Table

| Decision | Criteria |
| --- | --- |
| Included for ranking | Met all consensus criteria, for all participants and for individual professional groups |
| Included for re-rating | Met two consensus criteria and/or disagreement between groups (all participants, individual professional groups) |
| Removed From study | Met one or no consensus criteria, for all participants and for individual professional groups |

|  | All participants (n=31) | | | Pharmacist (n=14) | | | Physiotherapist (n=17) | | |
| --- | --- | --- | --- | --- | --- | --- | --- | --- | --- |
| Statement | Median | IQ range | % agreement | Median | IQ range | % agreement | Median | IQ range | % agreement |
| My knowledge of medication | 4 | 1 | 100 | 4 | 1 | 100 | 4 | 1 | 100 |
| Having a speciality allows development of skills and knowledge | 4 | 1 | 93.6 | 4 | 1 | 92.8 | 4 | 1 | 94.1 |
| Management support enables funding and training time to qualify as a prescriber | 5 | 1 | 90.3 | 5 | 1 | 85.7 | 5 | 1 | 94.1 |
| Patient requirements. A need for patient's to have streamlined care by being able to prescribe at the point of contact | 5 | 1 | 90.3 | 4 | 1 | 85.8 | 5 | 1 | 94.1 |
| Supportive medical colleagues | 4 | 1 | 90.3 | 4 | 1 | 92.8 | 4 | 1 | 88.3 |
| Being able to prescribe to patients is more effective and really useful working [in my area] | 5 | 1 | 87.1 | 5 | 1 | 92.9 | 5 | 1 | 82.4 |
| Easy access to medication info | 5 | 1 | 87.1 | 4.5 | 1 | 85.7 | 5 | 1 | 88.2 |
| Personal confidence in specialism | 4 | 0 | 87.1 | 4 | 1.25 | 78.6 | 4 | 0 | 94.1 |
| Supportive medical supervision / mentorship | 4 | 1 | 87.1 | 4 | 1.25 | 78.6 | 5 | 1 | 94.1 |
| The nature of the role facilitates prescribing practice as part of the overall review of patients | 4 | 1 | 87.1 | 4 | 1 | 92.9 | 5 | 1 | 82.3 |
| The law enables me to practice as an NMP | 4 | 1 | 87.1 | 4.5 | 1 | 92.9 | 4 | 1 | 82.4 |
| Motivation to help the patients who will benefit with prescribing and cut care delay / duplication | 4 | 1 | 87.1 | 4 | 1 | 85.8 | 5 | 1 | 88.2 |
| Working as part of an MDT [multidisciplinary team] / interdisciplinary group | 5 | 1 | 83.9 | 5 | 1.25 | 78.6 | 4 | 1 | 88.3 |
| Support from the employer/department for the role of non-medical prescribers | 4 | 1 | 83.9 | 4 | 1 | 85.7 | 5 | 1 | 82.3 |
| Supportive working environment [with NMP] policies in place | 4 | 1 | 83.9 | 4 | 2 | 71.4 | 4 | 1 | 94.1 |
| My employer has provided the support for me to be able to go on the NMP course and then supported me once qualified | 5 | 1 | 83.8 | 4 | 1 | 85.8 | 5 | 1 | 82.3 |
| Good relationship with consultants | 4 | 1 | 80.7 | 4.5 | 1 | 92.9 | 4 | 2 | 70.6 |
| Forward thinking DMP [designated medical practitioner] who is keen to integrate different MDG [multidisciplinary group] professionals into the team | 4 | 1 | 80.6 | 4 | 2 | 71.4 | 4 | 1 | 88.3 |
| Supportive nursing colleagues | 4 | 1 | 80.6 | 4 | 1 | 87.5 | 4 | 1.5 | 76.5 |
| Wide variety of options that you can offer patients to improve their experience | 4 | 1 | 80.6 | 4 | 1.25 | 71.4 | 5 | 1 | 88.2 |
| Well supported by team and they allow me to prescribe for their patients | 4 | 1 | 77.5 | 4.5 | 1 | 92.9 | 4 | 2 | 64.7 |
| Support from other NMPs | 4 | 1 | 77.4 | 4 | 1.25 | 78.6 | 4 | 1.5 | 76.5 |
| Clinical supervision with a [doctor] has massively helped me increase my confidence prescribing | 4 | 2 | 74.2 | 4 | 1.25 | 64.3 | 4 | 1 | 82.4 |
| Support from my line manager | 5 | 2 | 74.2 | 4.5 | 1 | 85.7 | 5 | 2.5 | 64.7 |
| Direct contact with medical team caring for patient | 4 | 2 | 74 | 4 | 1 | 85.7 | 4 | 1.5 | 82.4 |
| Doctors have been working [with] this [NMP] model | 4 | 1 | 71 | 4 | 1.5 | 71.4 | 4 | 1.5 | 70.5 |
| My manager is keen to develop non-medical prescribers within the trust so is supportive of my role and helping me to negotiate a clinic slot again. | 4 | 2 | 71 | 4 | 1.25 | 78.6 | 4 | 3 | 64.7 |
| Great antibiotic guidelines in this trust/area | 4 | 2 | 70.9 | 4 | 2 | 64.3 | 4 | 1.5 | 76.5 |
| [Benefit of] NICE Guidelines | 4 | 1 | 67.8 | 3.5 | 1 | 50 | 4 | 1 | 82.4 |
| Nursing and medical staff very open to pharmacist NMP role | 4 | 2 | 67.7 | 5 | 0.25 | 92.9 | 3 | 1 | 47.1 |
| When you see others doing, I think it gives you the confidence to do it yourself | 4 | 2 | 67.7 | 4 | 0.25 | 85.7 | 4 | 3 | 52.9 |
| Joint working / shadowing opportunities with the specialist prescribers or GPs | 4 | 2 | 67.7 | 4 | 2.25 | 71.5 | 4 | 2 | 64.7 |
| As an NMP I have much better knowledge of OTC [over the counter] medication and can advise patients accordingly | 4 | 2 | 67.7 | 3 | 2.25 | 42.8 | 4 | 1 | 88.2 |
| Mentor already NMP - creates a positive environment for NMP | 4 | 1 | 64.6 | 4 | 1.25 | 64.3 | 4 | 1 | 64.7 |
| Effective personal development reviews | 4 | 1 | 64.6 | 3 | 1.25 | 42.9 | 4 | 0 | 82.3 |
| Ongoing mentorship [supports] CPD [continuing professional development] and keeping up to date with current medication regimes | 4 | 2 | 64.5 | 3 | 1 | 42.9 | 4 | 1 | 82.4 |
| All patients rated the NMP experience as high and highly value their NMP prescribing as part of their care | 4 | 1 | 61.3 | 4 | 1 | 57.1 | 4 | 1.5 | 64.7 |
| My experience working as alongside a consultant/[GP] for many years | 4 | 1 | 61.3 | 4 | 1.5 | 78.5 | 3 | 1.5 | 47 |
| Evidence base from investigations | 4 | 1 | 61.3 | 4 | 1 | 57.1 | 4 | 1.5 | 64.7 |
| Attendance of MDT [multidisciplinary team] meeting [as] patients are discussed allowing the prescription to be discussed with the team | 4 | 2 | 61.3 | 4 | 1.25 | 64.3 | 4 | 2 | 58.8 |
| Clinical Lead pushing the project forwards | 4 | 1 | 58.1 | 4 | 1.25 | 78.6 | 3 | 1 | 70.6 |
| Supportive pharmacy leadership allowing prescribing without insisting on a second check by pharmacist | 4 | 2 | 58.1 | 4 | 2 | 57.2 | 4 | 2 | 58.8 |
| The department created a role that allows me to utilise my skillset and supports me in the role | 4 | 3 | 54.9 | 4 | 2.25 | 57.1 | 4 | 3 | 52.9 |
| Good NMP support group with regular meetings | 4 | 1 | 51.7 | 3 | 2 | 28.5 | 4 | 2 | 70.6 |
| Manager prompting [me] to do the course and plan how to introduce it in the department. | 4 | 2 | 51.7 | 4 | 2 | 71.2 | 3 | 2 | 35.3 |
| Having an electronic patient record mean that I can use all patient data available to base my prescribing upon | 4 | 2 | 51.6 | 3 | 2 | 42.9 | 4 | 2 | 58.9 |
| We are well supported with NMP training opportunities, including 2 full in-house training days a year | 3 | 2 | 48.4 | 3 | 2 | 35.7 | 4 | 1.5 | 58.8 |
| Process for registering, getting prescription pads etc... in place | 3 | 1 | 45.2 | 3 | 2.25 | 28.5 | 4 | 1 | 58.8 |
| Room space available for clinics | 3 | 2 | 38.7 | 3.5 | 1.5 | 50 | 3 | 2 | 29.4 |
| Prescribing regularly in primary care, a most advantageous skill | 3 | 2 | 35.5 | 3 | 1.25 | 14.2 | 4 | 1.5 | 52.9 |
| Being a role model for others [of my profession] as the only in-patient NMP | 3 | 2 | 35.5 | 3 | 1.5 | 21.4 | 3 | 1.5 | 47 |
| Electronic prescribing system allows an audit trail of my prescribing and pharmacists can easily access the prescriptions and verify them | 3 | 1 | 32.2 | 3 | 1.25 | 28.5 | 3 | 1 | 35.3 |
| Lack of medical cover proves the need have an extra prescriber on the ward | 3 | 1 | 29 | 3 | 1.5 | 42.9 | 3 | 0.5 | 17.7 |
| Medical colleagues informed by my frequent prescribing habits and have begun prescribing common drugs I often start a patient on | 3 | 2 | 29 | 3 | 2 | 28.6 | 3 | 2.5 | 29.4 |
| 30 years experience [giving confidence] | 3 | 1 | 19.4 | 3 | 2 | 14.2 | 3 | 1.5 | 23.5 |
| I have a [prescribing] budget where I work | 2 | 1 | 16.2 | 2 | 1 | 14.3 | 2 | 2 | 17.7 |
| As an IP [independent prescriber] it has made doing steroid injections much easier as less paperwork is necessary | 3 | 0 | 9.7 | 3 | 1.5 | 0 | 3 | 0.5 | 17.6 |
| As an IP [independent prescriber] it has made the process of steroid injections easier as I am able to mix medication | 3 | 0 | 9.7 | 3 | 0.5 | 0 | 3 | 0 | 17.6 |
| Nurses are not yet prescribers | 2 | 1 | 6.5 | 1.5 | 1.25 | 7.1 | 3 | 2 | 5.9 |
