# Supplementary material for: A Delphi study to explore and gain consensus regarding the most important barriers and facilitators affecting physiotherapist and pharmacist non-medical prescribing
Source: PLoS One. 2021 Feb 2;16(2):e0246273. doi: 10.1371/journal.pone.0246273 (PMC7853445; doi:10.1371/journal.pone.0246273)
Supplement: S3 Table — (DOCX) [file pone.0246273.s008.docx]

### S3 Table. Consensus results for Barrier statements, Round Two – grouped by all participants and for each profession

### Key to S3 Table

| Decision | Criteria |
| --- | --- |
| Included for ranking | Met all consensus criteria, for all participants and for individual professional groups |
| Included for re-rating | Met two consensus criteria and/or disagreement between groups (all participants, individual professional groups) |
| Removed From study | Met one or no consensus criteria, for all participants and for individual professional groups |

|  | All participants (n=31) | | | Pharmacist (n=14) | | | Physiotherapist (n=17) | | |
| --- | --- | --- | --- | --- | --- | --- | --- | --- | --- |
| Statement | Median | IQ range | % agreement | Median | IQ range | % agreement | Median | IQ range | % agreement |
| Lack of time to develop further prescribing skills | 4 | 1 | 67.7 | 4 | 1.25 | 71.4 | 4 | 1 | 64.7 |
| My confidence. I do sometimes doubt my abilities and worry a great deal about the legal/professional implications of making an incorrect decision | 4 | 2 | 67.8 | 4 | 2 | 57.1 | 4 | 0.5 | 76.4 |
| Lack of time to prescribe as core/clinical duties take priority | 4 | 3 | 54.8 | 4 | 3 | 64.3 | 3 | 2 | 47 |
| Lacking confidence as it is a new skill and not enough exposure | 4 | 2 | 51.6 | 2 | 3 | 42.8 | 4 | 2 | 76.4 |
| Skills learnt during NMP course cannot be put into practice until [professional] registration which took 2 months | 4 | 3 | 51.6 | 2.5 | 2.25 | 42.9 | 4 | 3 | 58.8 |
| Unable to prescribe [certain drugs] and have to ask a [doctor] to do this | 3 | 3 | 45.2 | 2 | 1.25 | 7.1 | 4 | 1.5 | 76.5 |
| Variable access to patient records. I would not be happy to prescribe when I did not have access to patient record with up to date medication/allergies etc. | 3 | 4 | 45.1 | 2 | 3.25 | 35.7 | 4 | 3 | 52.9 |
| Lack of training structure within the department[/workplace] | 3 | 2 | 42 | 3 | 1.25 | 21.4 | 4 | 2 | 58.8 |
| Keeping up with new research difficult | 3 | 2 | 41.9 | 3 | 2 | 42.8 | 3 | 2 | 41.2 |
| New ways of working from joining new team | 3 | 2 | 38.7 | 3 | 2 | 42.9 | 3 | 3 | 35.3 |
| Lack of diagnostic skills makes primary prescribing more difficult | 3 | 3 | 38.7 | 4 | 2 | 57.2 | 2 | 2.5 | 23.5 |
| Nurses are cheaper | 3 | 3 | 38.7 | 4 | 2.25 | 57.1 | 2 | 2.5 | 23.5 |
| NMP course very primary care and nursing focused | 3 | 3 | 35.5 | 1.5 | 2.25 | 21.4 | 3 | 3.5 | 47 |
| No identified prescribing role in current work area | 2 | 3 | 35.5 | 2 | 3 | 28.5 | 2 | 3.5 | 41.1 |
| Lack of suitable mentor/mentorship | 2 | 3 | 35.5 | 2 | 3 | 28.5 | 3 | 2.5 | 41.2 |
| Unable to access patient’s full SCR [summary care records] / GP records | 2 | 3 | 35.5 | 2 | 2.25 | 21.4 | 3 | 3.5 | 47 |
| No money to pay for staff going on any courses. However there seems to be a disparity as nurses in acute care appear to be able to access MSc modules | 2 | 3 | 35.5 | 2 | 2.25 | 21.4 | 3 | 2.5 | 47 |
| I am starting a new service, without much peer/managerial support to set it up | 3 | 2 | 32.3 | 2 | 2.25 | 28.5 | 3 | 2.5 | 35.3 |
| Starting a new speciality with new medicines to learn about | 3 | 3 | 32.3 | 3 | 2 | 35.7 | 3 | 3 | 29.4 |
| Lack of medical cover at times means I cannot prescribe opioids | 3 | 3 | 32.3 | 1 | 2 | 7.1 | 4 | 3 | 52.9 |
| [Lack of time] time available for prescribing activities. Facilitating attendance on ward round to allow full patient history and inpatient episode history | 3 | 3 | 32.3 | 4 | 2 | 57.1 | 2 | 2 | 11.8 |
| Nurse led clinic introducing nurse prescribers so no need for other prescribers | 3 | 3 | 32.3 | 3.5 | 2 | 50 | 2 | 2 | 17.7 |
| Lack of allotted time resulting from new management role | 3 | 3 | 32.3 | 3.5 | 2 | 50 | 3 | 2 | 17.6 |
| [Lack of] time to specialise | 2 | 2 | 32.3 | 2 | 2 | 28.6 | 3 | 2.5 | 35.3 |
| Clinical examination skills. Only basics taught on the course - BP and pulse. This makes me apprehensive to prescribe | 2 | 2 | 32.3 | 3.5 | 2.25 | 50 | 2 | 2 | 17.6 |
| I am unable to access any shared medical records making prescribing very difficult | 2 | 3 | 32.3 | 1 | 3 | 28.6 | 3 | 3.5 | 35.3 |
| NMP role not well established for [my profession] | 2 | 3 | 32.3 | 2 | 2 | 14.3 | 3 | 2.5 | 47 |
| Lack of communication from university following course completion | 2 | 3 | 32.3 | 2 | 1.5 | 21.4 | 3 | 3 | 41.2 |
| Lack of clinic rooms | 2 | 3 | 32.3 | 3.5 | 3.25 | 50 | 2 | 2 | 17.6 |
| Poor integration between the community team and the hospital team | 3 | 2 | 32.2 | 3 | 3 | 28.6 | 3 | 1.5 | 35.2 |
| Secondary care outpatient specialities should suggest course of action to the GP regarding medicines. (Clinical Pathways and Hospital Policies) | 3 | 2 | 29.1 | 3 | 1.25 | 21.4 | 3 | 1.5 | 35.3 |
| [Unable to prescribe certain drugs and have to use] supplementary prescribing, [which] requires a slight change to the pathway of the team and doctors need to be educated | 2 | 3 | 29.1 | 1 | 1.25 | 7.1 | 3 | 3 | 47.1 |
| Limitations of [legal] prescribing guidelines [with a disparity between practitioner roles] | 3 | 3 | 29 | 1.5 | 1 | 7.1 | 3 | 2 | 47 |
| Lack of access to ongoing development out of Trust | 2 | 2 | 29 | 3 | 1.25 | 21.4 | 2 | 2.5 | 35.3 |
| The availability of a pharmacist to clinically screen the prescriptions | 2 | 3 | 25.9 | 2 | 2.25 | 42.9 | 2 | 1.5 | 11.8 |
| Cost of professional indemnity | 2 | 3 | 25.9 | 2.5 | 2.25 | 35.7 | 2 | 1.5 | 17.79 |
| Professional indemnity is a challenge to acquire - need updated JD [job description] and employer slow to produce | 3 | 2 | 22.6 | 2 | 2.25 | 21.4 | 3 | 1.5 | 23.6 |
| There is no time to actively prescribe [in my current role] | 2 | 3 | 25.9 | 2 | 3 | 28.6 | 2 | 2.5 | 23.5 |
| Changed roles [and] I don't feel confident to prescribe in the area that I work in now | 2 | 3 | 25.9 | 2 | 2.25 | 21.4 | 3 | 2.5 | 29.4 |
| There is no current budget for prescribing | 3 | 2 | 25.8 | 3 | 2 | 14.3 | 3 | 2 | 35.2 |
| Pressure from ward to prescribe beyond my scope | 3 | 3 | 25.8 | 3 | 2.25 | 28.5 | 3 | 2.5 | 23.6 |
| Due to change employment shortly where new post doesn't currently have prescribing for [my profession] in place | 3 | 3 | 25.8 | 2 | 2.25 | 21.4 | 3 | 2.5 | 29.4 |
| Non-attendance on clinical ward rounds as documentation of patient progress or clinical plan in notes not always clear | 3 | 1 | 22.6 | 3 | 2 | 35.7 | 3 | 2 | 11.8 |
| Change in job role, [to one that] did not lend itself to prescribing | 3 | 1 | 22.6 | 2 | 1.5 | 21.4 | 3 | 2 | 23.6 |
| Gaining permission for pilot and initial funding for clinics | 3 | 1 | 22.6 | 3 | 2 | 28.5 | 3 | 2 | 17.7 |
| Formulary differences between Trust and APC/CCG [area prescribing committee/clinical commissioning group] make it difficult to know what I can prescribe | 3 | 2 | 22.6 | 2 | 2 | 14.3 | 3 | 2 | 29.4 |
| The department is not very supportive within the context of expanding my role and utilising the practical aspects of my prescribing such as patient examination | 2 | 1 | 22.6 | 2 | 1.25 | 21.4 | 2 | 2.5 | 23.6 |
| A lack of clinicians wanting to share their skills | 2 | 2 | 22.6 | 2 | 2.25 | 21.4 | 2 | 2.5 | 23.5 |
| Lack of organisational funding | 2 | 2 | 22.6 | 2 | 1.25 | 14.2 | 3 | 3 | 29.4 |
| Managers not supporting prescribing role | 2 | 2 | 22.6 | 2 | 1 | 14.2 | 2 | 2.5 | 29.4 |
| Lack of staffing, so often conducting medicines reconciliation, which I am reluctant to prescribe from, and may not have time to go on the ward round | 3 | 1 | 19.4 | 3 | 2 | 42.9 | 2 | 2 | 0 |
| You've had to stop a particular drug, even though licensed for reason being prescribed, as CCG [clinical commissioning group] is following guideline | 3 | 1 | 19.4 | 2.5 | 1 | 14.3 | 3 | 2 | 23.6 |
| [My] prescribing not reviewed by pharmacists in the same way as medic or other NMPs prescribing | 2 | 2 | 19.4 | 2 | 2.25 | 21.4 | 2 | 2 | 17.7 |
| Lack of clear requirements to what is competent | 2 | 2 | 19.4 | 2 | 1.5 | 21.4 | 2 | 2 | 17.6 |
| Lack of acceptance by [medics] | 2 | 2 | 19.4 | 2 | 1.25 | 14.3 | 2 | 2.5 | 23.5 |
| Lack of immediate medical advice/support | 2 | 2 | 19.3 | 2 | 2 | 14.2 | 2 | 2.5 | 23.5 |
| Lack of pharmacology exposure during undergraduate training | 2 | 2 | 19.3 | 1 | 1 | 0 | 3 | 3 | 35.3 |
| [Lack of] Funding for time spent prescribing | 2 | 2 | 19.3 | 2 | 1.5 | 21.4 | 2 | 2 | 17.7 |
| I have no other IP [independent prescriber] to chat things through with quickly & easily | 1 | 1 | 19.3 | 1 | 1 | 7.1 | 2 | 3 | 29.4 |
| Community/lone working [as] we only ever see patients on our own, so I'm unable to gain advice from other sources immediately | 3 | 1 | 16.2 | 3 | 1.25 | 7.1 | 3 | 1.5 | 23.6 |
| Trust application process lengthy and still waiting to be allowed to prescribe. Director of nursing does not know me and has been reluctant to sign paperwork | 2 | 2 | 16.2 | 1.5 | 1 | 14.2 | 2 | 2 | 17.7 |
| Sometimes junior clinicians feel an NMP is prescribing because their own prescribing is inadequate | 3 | 2 | 16.1 | 1.5 | 2.25 | 21.4 | 3 | 1.5 | 11.8 |
| Colleagues may feel prescribing should only occur after all the usual duties have been completed | 2 | 2 | 16.1 | 3 | 2.25 | 35.7 | 2 | 2 | 0 |
| [Lack of] a defined reason to prescribe | 2 | 2 | 16.1 | 2 | 2.25 | 21.4 | 2 | 2 | 11.8 |
| [Medic] instead of increasing the dose may change the drug instead of discussing with me | 2 | 2 | 16.1 | 2 | 2 | 14.3 | 3 | 2 | 17.6 |
| [Lack of] acceptance as NMP by nurses | 1 | 2 | 16.1 | 1 | 1 | 7.1 | 2 | 2.5 | 17.6 |
| NMP in clinic is not recognised as not commissioned | 3 | 2 | 9.7 | 3 | 1 | 7.1 | 3 | 2 | 11.8 |
| Rely on external company to register NMPs, print pads etc... sometimes delays someone being able to prescribe | 1 | 2 | 6.5 | 1 | 2 | 0 | 1 | 2 | 11.8 |
