# Supplementary material for: A Delphi study to explore and gain consensus regarding the most important barriers and facilitators affecting physiotherapist and pharmacist non-medical prescribing
Source: PLoS One. 2021 Feb 2;16(2):e0246273. doi: 10.1371/journal.pone.0246273 (PMC7853445; doi:10.1371/journal.pone.0246273)
Supplement: S4 Table — (DOCX) [file pone.0246273.s009.docx]

**S4 Table. Consensus results for Facilitator statements, Round Three – grouped by all participants and for each profession. Round Two results included for comparison (see key)**

Key:

| Rd 2 = Round Two results | All participants (n=31) | Pharmacist (n=14) | Physiotherapist (n=17) |
| --- | --- | --- | --- |
| Rd 3 = Round Three results | All participants (n=20) | Pharmacist (n=10) | Physiotherapist (n=10) |

| **Statement** | **All participants** | | | | | | **Pharmacists** | | | | | | **Physiotherapists** | | | | | |
| --- | --- | --- | --- | --- | --- | --- | --- | --- | --- | --- | --- | --- | --- | --- | --- | --- | --- | --- |
|  | **Median** | | **IQ range** | | **% agreement** | | **Median** | | **IQ range** | | **% agreement** | | **Median** | | **IQ range** | | **% agreement** | |
|  | Rd 2 | Rd 3 | Rd 2 | Rd 3 | Rd 2 | Rd 3 | Rd 2 | Rd 3 | Rd 2 | Rd 3 | Rd 2 | Rd 3 | Rd 2 | Rd 3 | Rd 2 | Rd 3 | Rd 2 | Rd 3 |
| When you see others doing, I think it gives you the confidence to do it yourself | 4 | 4 | 2 | 0 | 67.7 | 80 | 4 | 4 | 0.25 | 0 | 85.7 | 90 | 4 | 4 | 3 | 1.25 | 52.9 | 70 |
| Nursing and medical staff very open to pharmacist NMP role | 4 | 4.5 | 2 | 1.75 | 67.7 | 75 | 5 | 5 | 0.25 | 0 | 92.9 | 90 | 3 | 4 | 1 | 1.5 | 47.1 | 60 |
| Ongoing mentorship [supports] CPD [continuing professional development] and keeping up to date with current medication regimes | 4 | 4 | 2 | 1.75 | 64.5 | 75 | 3 | 4 | 1 | 1.25 | 42.9 | 60 | 4 | 5 | 1 | 1 | 82.4 | 90 |
| [Benefit of] NICE Guidelines | 4 | 4 | 1 | 1 | 67.8 | 70 | 3.5 | 4 | 1 | 1 | 50 | 60 | 4 | 4 | 1 | 1.5 | 82.4 | 80 |
| Effective personal development reviews | 4 | 4 | 1 | 1 | 64.6 | 70 | 3 | 4 | 1.25 | 1 | 42.9 | 60 | 4 | 4 | 0 | 1.25 | 82.3 | 80 |
| Joint working / shadowing opportunities with the specialist prescribers or GPs | 4 | 4 | 2 | 2 | 67.7 | 65 | 4 | 4 | 2.25 | 1.25 | 71.5 | 80 | 4 | 3.5 | 2 | 3 | 64.7 | 50 |
| Supportive pharmacy leadership allowing prescribing without insisting on a second check by pharmacist | 4 | 4 | 2 | 2.75 | 58.1 | 65 | 4 | 4 | 2 | 1.5 | 57.2 | 80 | 4 | 3.5 | 2 | 4 | 58.8 | 50 |
| My experience working as alongside a consultant/[GP] for many years | 4 | 4 | 1 | 1.75 | 61.3 | 60 | 4 | 4 | 1.5 | 1.25 | 78.5 | 70 | 3 | 3.5 | 1.5 | 2.5 | 47 | 50 |
| Attendance of MDT [multidisciplinary team] meeting [as] patients are discussed allowing the prescription to be discussed with the team | 4 | 4 | 2 | 1.75 | 61.3 | 60 | 4 | 4 | 1.25 | 3 | 64.3 | 60 | 4 | 4 | 2 | 1.25 | 58.8 | 60 |
| All patients rated the NMP experience as high and highly value their NMP prescribing as part of their care | 4 | 4 | 1 | 1 | 61.3 | 55 | 4 | 3.5 | 1 | 1 | 57.1 | 55 | 4 | 4 | 1.5 | 1.25 | 64.7 | 60 |
| Good NMP support group with regular meetings | 4 | 4 | 1 | 1 | 51.7 | 55 | 3 | 3 | 2 | 1.25 | 28.5 | 40 | 4 | 4 | 2 | 2 | 70.6 | 70 |
| Manager prompting [me] to do the course and plan how to introduce it in the department. | 4 | 4 | 2 | 1.75 | 51.7 | 55 | 4 | 5 | 2 | 2 | 71.2 | 70 | 3 | 3.5 | 2 | 1.25 | 35.3 | 40 |
| My manager is keen to develop non-medical prescribers within the trust so is supportive of my role and helping me to negotiate a clinic slot again. | 4 | 4 | 2 | 2 | 71 | 55 | 4 | 4 | 1.25 | 2.25 | 78.6 | 70 | 4 | 3 | 3 | 2 | 64.7 | 40 |
| Evidence base from investigations | 4 | 3.5 | 1 | 1 | 61.3 | 50 | 4 | 3 | 1 | 1 | 57.1 | 30 | 4 | 4 | 1.5 | 1.25 | 64.7 | 70 |
| As an NMP I have much better knowledge of OTC [over the counter] medication and can advise patients accordingly | 4 | 3.5 | 2 | 1 | 67.7 | 50 | 3 | 3 | 2.25 | 1 | 42.8 | 10 | 4 | 4 | 1 | 1 | 88.2 | 90 |
| Clinical Lead pushing the project forwards | 4 | 3.5 | 1 | 1 | 58.1 | 50 | 4 | 3.5 | 1.25 | 1.25 | 78.6 | 50 | 3 | 3.5 | 1 | 1.75 | 70.6 | 50 |
| Having an electronic patient record mean that I can use all patient data available to base my prescribing upon | 4 | 3 | 2 | 2 | 51.6 | 45 | 3 | 3 | 2 | 1.25 | 42.9 | 30 | 4 | 4 | 2 | 2 | 58.9 | 60 |
| We are well supported with NMP training opportunities, including 2 full in-house training days a year | 3 | 3 | 2 | 1.75 | 48.4 | 45 | 3 | 3 | 2 | 2 | 35.7 | 40 | 4 | 3.5 | 1.5 | 1.5 | 58.8 | 50 |
| Process for registering, getting prescription pads etc... in place | 3 | 3 | 1 | 1 | 45.2 | 30 | 3 | 3 | 2.25 | 1 | 28.5 | 30 | 4 | 3 | 1 | 1.25 | 58.8 | 30 |
| Prescribing regularly in primary care, a most advantageous skill | 3 | 3 | 2 | 1 | 35.5 | 30 | 3 | 3 | 1.25 | 0 | 14.2 | 10 | 4 | 3.5 | 1.5 | 2 | 52.9 | 50 |
| Medical colleagues informed by my frequent prescribing habits and have begun prescribing common drugs I often start a patient on | 3 | 3 | 2 | 2 | 29 | 30 | 3 | 3 | 2 | 1.25 | 28.6 | 20 | 3 | 3.5 | 2.5 | 2 | 29.4 | 40 |
