# Supplementary material for: A Delphi study to explore and gain consensus regarding the most important barriers and facilitators affecting physiotherapist and pharmacist non-medical prescribing
Source: PLoS One. 2021 Feb 2;16(2):e0246273. doi: 10.1371/journal.pone.0246273 (PMC7853445; doi:10.1371/journal.pone.0246273)
Supplement: S5 Table — (DOCX) [file pone.0246273.s010.docx]

**S5 Table. Consensus results for Barrier statements, Round Three – grouped by all participants and for each profession. Round Two results included for comparison (see key)**

Key:

| Rd 2 = Round Two results | All participants (n=31) | Pharmacist (n=14) | Physiotherapist (n=17) |
| --- | --- | --- | --- |
| Rd 3 = Round Three results | All participants (n=20) | Pharmacist (n=10) | Physiotherapist (n=10) |

| **Statement** | **All participants** | | | | | | **Pharmacists** | | | | | | **Physiotherapists** | | | | | |
| --- | --- | --- | --- | --- | --- | --- | --- | --- | --- | --- | --- | --- | --- | --- | --- | --- | --- | --- |
|  | **Median** | | **IQ range** | | **% agreement** | | **Median** | | **IQ range** | | **% agreement** | | **Median** | | **IQ range** | | **% agreement** | |
|  | Rd 2 | Rd 3 | Rd 2 | Rd 3 | Rd 2 | Rd 3 | Rd 2 | Rd 3 | Rd 2 | Rd 3 | Rd 2 | Rd 3 | Rd 2 | Rd 3 | Rd 2 | Rd 3 | Rd 2 | Rd 3 |
| My confidence. I do sometimes doubt my abilities and worry a great deal about the legal/professional implications of making an incorrect decision | 4 | 4 | 2 | 2 | 67.8 | 60 | 4 | 4 | 2 | 2 | 57.1 | 70 | 4 | 3.5 | 0.5 | 2 | 76.4 | 50 |
| Skills learnt during NMP course cannot be put into practice until [professional] registration which took 2 months | 4 | 4 | 3 | 2 | 51.6 | 60 | 2.5 | 3.5 | 2.25 | 2.5 | 42.9 | 50 | 4 | 4 | 3 | 1.5 | 58.8 | 70 |
| Lack of time to prescribe as core/clinical duties take priority | 4 | 4 | 3 | 3 | 54.8 | 60 | 4 | 4.5 | 3 | 1.5 | 64.3 | 80 | 3 | 2.5 | 2 | 2.25 | 47 | 40 |
| Lacking confidence as it is a new skill and not enough exposure | 4 | 3 | 2 | 2 | 51.6 | 45 | 2 | 3 | 3 | 2.25 | 42.8 | 40 | 4 | 3.5 | 2 | 2.25 | 76.4 | 50 |
| Lack of allotted time resulting from new management role | 3 | 3 | 3 | 1.75 | 32.3 | 45 | 3.5 | 4 | 2 | 1.25 | 50 | 80 | 3 | 2.5 | 2 | 2 | 17.6 | 10 |
| Variable access to patient records. I would not be happy to prescribe when I did not have access to patient record with up to date medication/allergies etc. | 3 | 3 | 4 | 3 | 45.1 | 45 | 2 | 4.5 | 3.25 | 2.25 | 35.7 | 60 | 4 | 3 | 3 | 2.25 | 52.9 | 30 |
| Unable to prescribe [certain drugs] and have to ask a [doctor] to do this | 3 | 3 | 3 | 2 | 45.2 | 40 | 2 | 2 | 1.25 | 1.25 | 7.1 | 0 | 4 | 4 | 1.5 | 1.25 | 76.5 | 80 |
| Lack of diagnostic skills makes primary prescribing more difficult | 3 | 3 | 3 | 2.75 | 38.7 | 40 | 4 | 4 | 2 | 1.5 | 57.2 | 70 | 2 | 2 | 2.5 | 2 | 23.5 | 10 |
| [Lack of time] time available for prescribing activities. Facilitating attendance on ward round to allow full patient history and inpatient episode history | 3 | 3 | 3 | 2.75 | 32.3 | 40 | 4 | 4 | 2 | 3 | 57.1 | 60 | 2 | 3 | 2 | 3 | 11.8 | 20 |
| I am starting a new service, without much peer/managerial support to set it up | 3 | 3 | 2 | 2 | 32.3 | 35 | 2 | 2.5 | 2.25 | 2 | 28.5 | 40 | 3 | 3 | 2.5 | 2 | 35.3 | 30 |
| Starting a new speciality with new medicines to learn about | 3 | 3 | 3 | 2.75 | 32.3 | 35 | 3 | 3.5 | 2 | 2.25 | 35.7 | 50 | 3 | 3 | 3 | 2.25 | 29.4 | 20 |
| Lack of communication from university following course completion | 2 | 2.5 | 3 | 2 | 32.3 | 35 | 2 | 2 | 1.5 | 3 | 21.4 | 40 | 3 | 3 | 3 | 2.25 | 41.2 | 30 |
| [Lack of] time to specialise | 2 | 2 | 2 | 3 | 32.3 | 35 | 2 | 2.5 | 2 | 3 | 28.6 | 40 | 3 | 2 | 2.5 | 3 | 35.3 | 30 |
| Nurses are cheaper | 3 | 3 | 3 | 2.75 | 38.7 | 30 | 4 | 3 | 2.25 | 2.5 | 57.1 | 40 | 2 | 2.5 | 2.5 | 2.25 | 23.5 | 20 |
| Nurse led clinic introducing nurse prescribers so no need for other prescribers | 3 | 3 | 3 | 2.75 | 32.3 | 30 | 3.5 | 3.5 | 2 | 2.25 | 50 | 50 | 2 | 2 | 2 | 2 | 17.7 | 10 |
| Lack of access to ongoing development out of Trust | 2 | 2 | 2 | 2 | 29 | 30 | 3 | 2 | 1.25 | 2.25 | 21.4 | 30 | 2 | 2 | 2.5 | 2.25 | 35.3 | 30 |
| Lack of immediate medical advice/support | 2 | 2 | 2 | 2 | 19.3 | 30 | 2 | 2 | 2 | 0.5 | 14.2 | 20 | 2 | 2 | 2.5 | 3 | 23.5 | 40 |
| [Lack of] a defined reason to prescribe | 2 | 2 | 2 | 3 | 16.1 | 30 | 2 | 2 | 2.25 | 3 | 21.4 | 40 | 2 | 2 | 2 | 2.25 | 11.8 | 20 |
| Poor integration between the community team and the hospital team | 3 | 3 | 2 | 1.5 | 32.2 | 25 | 3 | 3 | 3 | 2.5 | 28.6 | 30 | 3 | 3 | 1.5 | 0.75 | 35.2 | 20 |
| Lack of clinic rooms | 2 | 3 | 3 | 1.75 | 32.3 | 25 | 3.5 | 3 | 3.25 | 2.25 | 50 | 40 | 2 | 2 | 2 | 1.25 | 17.6 | 10 |
| Lack of medical cover at times means I cannot prescribe opioids | 3 | 3 | 3 | 2.75 | 32.3 | 25 | 1 | 2 | 2 | 2 | 7.1 | 10 | 4 | 3 | 3 | 1.75 | 52.9 | 40 |
| Lack of organisational funding | 2 | 2 | 2 | 1.75 | 22.6 | 25 | 2 | 2 | 1.25 | 2 | 14.2 | 30 | 3 | 2 | 3 | 2.25 | 29.4 | 20 |
| The department is not very supportive within the context of expanding my role and utilising the practical aspects of my prescribing such as patient examination | 2 | 2 | 1 | 2.75 | 22.6 | 25 | 2 | 2 | 1.25 | 2.25 | 21.4 | 20 | 2 | 2 | 2.5 | 3 | 23.6 | 30 |
| New ways of working from joining new team | 3 | 3 | 2 | 1 | 38.7 | 20 | 3 | 3 | 2 | 1.25 | 42.9 | 30 | 3 | 3 | 3 | 2 | 35.3 | 10 |
| Limitations of [legal] prescribing guidelines [with a disparity between practitioner roles] | 3 | 3 | 3 | 1.75 | 29 | 15 | 1.5 | 2 | 1 | 2 | 7.1 | 0 | 3 | 3 | 2 | 2.5 | 47 | 30 |
| Cost of professional indemnity | 2 | 2 | 3 | 1 | 25.9 | 20 | 2.5 | 2.5 | 2.25 | 2 | 35.7 | 40 | 2 | 2 | 1.5 | 1 | 17.8 | 0 |
| NMP role not well established for [my profession] | 2 | 2 | 3 | 1 | 32.3 | 20 | 2 | 2 | 2 | 2 | 14.3 | 10 | 3 | 2 | 2.5 | 2 | 47 | 30 |
| The availability of a pharmacist to clinically screen the prescriptions | 2 | 2 | 3 | 1.75 | 25.9 | 20 | 2 | 3 | 2.25 | 2.25 | 42.9 | 40 | 2 | 2 | 1.5 | 1 | 11.8 | 0 |
| A lack of clinicians wanting to share their skills | 2 | 2 | 2 | 1.75 | 22.6 | 20 | 2 | 2 | 2.25 | 1.25 | 21.4 | 10 | 2 | 2.5 | 2.5 | 3 | 23.5 | 30 |
| [My] prescribing not reviewed by pharmacists in the same way as medic or other NMPs prescribing | 2 | 2 | 2 | 1.75 | 19.4 | 20 | 2 | 2 | 2.25 | 3 | 21.4 | 30 | 2 | 2 | 2 | 1 | 17.7 | 10 |
| Colleagues may feel prescribing should only occur after all the usual duties have been completed | 2 | 2 | 2 | 1.75 | 16.1 | 20 | 3 | 2.5 | 2.25 | 2.25 | 35.7 | 40 | 2 | 2 | 2 | 2 | 0 | 0 |
| [Unable to prescribe certain drugs and have to use] supplementary prescribing, [which] requires a slight change to the pathway of the team and doctors need to be educated | 2 | 2 | 3 | 2 | 29.1 | 20 | 1 | 1.5 | 1.25 | 1.25 | 7.1 | 10 | 3 | 2 | 3 | 3.25 | 47.1 | 30 |
| Lack of training structure within the department[/workplace] | 3 | 2.5 | 2 | 1 | 42 | 15 | 3 | 3 | 1.25 | 1.25 | 21.4 | 20 | 4 | 2 | 2 | 2 | 58.8 | 10 |
| I have no other IP [independent prescriber] to chat things through with quickly & easily | 1 | 1 | 1 | 1 | 19.3 | 15 | 1 | 1 | 1 | 1 | 7.1 | 10 | 2 | 2 | 3 | 2.25 | 29.4 | 20 |
| Professional indemnity is a challenge to acquire - need updated JD [job description] and employer slow to produce | 3 | 2.5 | 2 | 1.75 | 22.6 | 10 | 2 | 2 | 2.25 | 2 | 21.4 | 10 | 3 | 3 | 1.5 | 1.25 | 23.6 | 10 |
| Lack of pharmacology exposure during undergraduate training | 2 | 2 | 2 | 2 | 19.3 | 10 | 1 | 1.5 | 1 | 2 | 0 | 0 | 3 | 2 | 3 | 1.5 | 35.3 | 20 |
| Sometimes junior clinicians feel an NMP is prescribing because their own prescribing is inadequate | 3 | 2 | 2 | 2 | 16.1 | 10 | 1.5 | 2 | 2.25 | 2.25 | 21.4 | 20 | 3 | 2.5 | 1.5 | 2 | 11.8 | 0 |
