# Supplementary material for: A Delphi study to explore and gain consensus regarding the most important barriers and facilitators affecting physiotherapist and pharmacist non-medical prescribing
Source: PLoS One. 2021 Feb 2;16(2):e0246273. doi: 10.1371/journal.pone.0246273 (PMC7853445; doi:10.1371/journal.pone.0246273)
Supplement: Attachment — Submitted filename: Response to Reviewers.docx [file pone.0246273.s011.docx]

Vijayaprakash Suppiah, PhD

Academic Editor

PLOS ONE

PONE-D-20-27040

Dear Dr Suppiah

**Re: Response to Reviewers - A Delphi study to explore and gain consensus regarding the most important barriers and facilitators affecting physiotherapist and pharmacist non-medical prescribing**

Thank you for the feedback on our manuscript and the opportunity to submit a revised manuscript, addressing the reviewer’s comments. As requested, we submit a revised version, complete with tracked changes, and a complete manuscript.

The table below outlines the changes made in response to the reviewer’s comments.

All authors have approved the edited manuscript and submission to PLOS ONE. We appreciate your time and thank you for considering this manuscript for publication. Should you have any further questions please do not hesitate to contact us.

Yours sincerely

Emma Graham-Clarke MPhil, BPharm, FRPharmS, FFRPS

On behalf of the authors

| **Editor’s Comments** | **Line numbers affected in original manuscript** | **Authors Response** |
| --- | --- | --- |
| Please ensure that your manuscript meets PLOS ONE's style requirements, including those for file naming. |  | *We have checked the manuscript and amended it where necessary.*  *Additional supporting information files have been appropriately named* |
| Please include additional information regarding the questionnaires used in the study and ensure that you have provided sufficient details that others could replicate the analyses. For instance, if you developed a questionnaire as part of this study and it is not under a copyright more restrictive than CC-BY, please include a copy, in both the original language and English, as Supporting Information. |  | *We have included copies of all the questionaries as supporting information (S2, S3 and S4 Appendices).*  *The consensus results for Round Three have been added as supporting information to enable comparison with the Round Two results (S4 and S5 Tables)*  *A worked example of the weighting for ranked results in Round Three has been added as supporting information (S5 Appendix) to clarify the process.* |
| **Reviewer’s Comments** | **Line numbers affected in original manuscript** | **Authors Response** |
| Thank you for the opportunity to review this manuscript exploring NMP in allied health. The manuscript is a useful addition to the literature. The Introduction and Methods are well described and easy to follow. |  | *Thank you for your kind comments and feedback.* |
| Please describe how Kendall's w was interpreted. | 167 | *The criteria for interpreting Kendall’s Coefficient of Concordance (W) are included in Table 1, and we have added an additional sentence to clarify the potential range of results for Kendall’s Coefficient of Concordance (W).* |
| Table 5 - please amend the p-values to <0.01 or similar. p-values can't be 0. | 232, 265, 280 | *Thank you for highlighting this. We have amended Tables 5, 6 and 7 accordingly and also amended the column heading to further clarify the tables.* |
| It may be helpful to focus the initial aspects of the Discussion on the results that were obtained. Currently, the whole Discussion reads as the 'strengths and limitations' although I appreciate the thoroughness with which the authors have considered these issues. | 297 et seq | *We have reordered the Discussion as you suggest, highlighting the initial findings.* |
